# The effectiveness of improving healthcare teams’ human factor skills using simulation-based training: a systematic review

**DOI:** 10.1186/s41077-022-00207-2

**Published:** 2022-05-07

**Authors:** Lotte Abildgren, Malte Lebahn-Hadidi, Christian Backer Mogensen, Palle Toft, Anders Bo Nielsen, Tove Faber Frandsen, Sune Vork Steffensen, Lise Hounsgaard

**Affiliations:** 1 Anesthesiology and Intensive Care Unit, Odense University Hospital, Odense, Denmark; 2 OPEN, Open Patient data Explorative Network, Odense University Hospital/Department of Clinical Research, University of Southern Denmark, Odense, Denmark; 3 Emergency Research Unit, Hospital Sønderjylland, University Hospital of Southern Denmark, Odense, Denmark; 4 Centre for Human Interactivity, Department of Language and Communication, University of Southern Denmark, Odense, Denmark; 5 Department of Clinical Research, University of Southern Denmark, Odense, Denmark; 6 SimC, Regional Center for Technical Simulation, Region of Southern Denmark, Odense, Denmark; 7 Department of Design and Communication, University of Southern Denmark, Kolding, Denmark; 8 Danish Institute for Advanced Study, University of Southern Denmark, Odense, Denmark; 9 Center for Ecolinguistics, South China Agricultural University, Guangzhou, People’s Republic of China; 10 College of International Studies, Southwest University, Chongqing, People’s Republic of China; 11 Institute of Nursing & Health Science, Ilisimartusarfik, University of Greenland, Nuuk, Greenland; 12 Center for Mental Health Nursing and Health Research (CPS), Mental Health Services, Region of Southern Denmark, University of Southern Denmark, Odense, Denmark

**Keywords:** Systematic review, Simulation-based training, Medical simulation, Human factor skills, Non-technical skills (NTS), Adverse events, Teamwork, Crisis resource management (CRM), Qualified healthcare team, In-hospital

## Abstract

**Background:**

Simulation-based training used to train healthcare teams’ skills and improve clinical practice has evolved in recent decades. While it is evident that technical skills training is beneficial, the potential of human factor training has not been described to the same extent. Research on human factor training has been limited to marginal and acute care scenarios and often to validate instruments. This systematic review aimed to investigate the effectiveness of simulation-based training in improving in-hospital qualified healthcare teams’ human factor skills.

**Method:**

A review protocol outlining the study was registered in PROSPERO. Using the PRISMA guidelines, the systematic search was conducted on September 28th, 2021, in eight major scientific databases. Three independent reviewers assessed title and abstract screening; full texts were evaluated by one reviewer. Content analysis was used to evaluate the evidence from the included studies.

**Results:**

The search yielded 19,767 studies, of which 72 were included. The included studies were published between 2004 and 2021 and covered research from seven different in-hospital medical specialisms. Studies applied a wide range of assessment tools, which made it challenging to compare the effectiveness of human factor skills training across studies. The content analysis identified evidence for the effectiveness. Four recurring themes were identified: (1) Training human factor skills in qualified healthcare teams; (2) assessment of human factor skills; (3) combined teaching methods, and (4) retention and transfer of human factor skills. Unfortunately, the human factor skills assessments are variable in the literature, affecting the power of the result.

**Conclusion:**

Simulation-based training is a successful learning tool to improve qualified healthcare teams’ human factor skills. Human factor skills are not innate and appear to be trainable similar to technical skills, based on the findings of this review. Moreover, research on retention and transfer is insufficient. Further, research on the retention and transfer of human factor skills from simulation-based training to clinical practice is essential to gain knowledge of the effect on patient safety.

**Supplementary Information:**

The online version contains supplementary material available at 10.1186/s41077-022-00207-2.

## Background

Adverse events^1^ are common in hospitals all over the world. They cause higher mortality and morbidity, along with more pain and increased healthcare costs [1]. Since 2004, the number of reported adverse events in Denmark has increased and has stabilised at a relatively high level [2]. The Danish Patient Safety Strategy [3] has an organisational approach that addresses adverse events by providing knowledge through guidelines, e-learning, and newsletters [4, 5]. Providing knowledge implies that adverse events might be avoided through enhanced guidelines and safety procedures. However, several studies find that adverse events often occur in non-routine, complex environments due to interactions between humans and the systems in which they work. These interactions are modifiable due to learning skills (e.g. leadership-followership, decision-making and coordination) rather than lack of knowledge [6–9]. The medical simulation and patient safety literature most often refer to these aspects as non-technical skills, crisis resource management or interpersonal relations [9–14]. These common concepts are too limited, however, since they specifically define competence in terms of what is lacking (non-technical skills), what it is for (crises resource management) or interaction between people (interpersonal relations). The comprehensive concept of human factors includes broader aspects of human interaction, including social skills, cognitive skills and decision-making. It emphasises how the environment, the organisation and human psychology interact [15, 16]. Based on this reflection, this article will use human factors skills (HFS) as the terminology for the skills in focus. Patient safety reports and root cause analysis indicate that adverse events occur in interactions between technology, organisation and human factors, and adverse events are about understanding the interactions among humans and other elements of a system, including social and cognitive structures [1, 2, 17]. An example is the relocation of healthcare personnel from their everyday work to COVID-19 units [18]. This challenged even highly competent personnel and might have caused an increased number of human errors. Personnel had to adapt to unfamiliar technical and cognitive procedures and new surroundings, complications, colleagues and workflows. The Danish Patient Safety Database shows a 32% increase in reported adverse events in 2020 [19], with a peak at the beginning of the COVID-19 pandemic.

Research indicates that simulation-based training (SBT) is a safe and effective tool to develop and increase competencies in healthcare [20]. However, existing reviews focus on technical skills (TS), self-confidence, self-efficacy and the effectiveness of SBT for unqualified healthcare students [21–24] and develop unqualified healthcare students’ HFS [25, 26]. SBT has been found to refine qualified healthcare teams’ TS, self-efficacy and confidence [24, 27]. Existing studies of qualified healthcare teams’ HFS focus on developing curricula, specific settings or situations or testing new evaluation or rating instruments [28–32]. Buljac-Samardzic et al. [33] explored interventions that improved team effectiveness and concluded that SBT enhances teamwork, though interventions studies were limited to specific situations, settings and outcomes. As mentioned, HFS are crucial to reducing adverse events [34], but evidence concerning the effectiveness of SBT to refine qualified healthcare teams’ use of HFS is sparse. There is a need for additional knowledge about the effectiveness of developing HFS in qualified healthcare teams with SBT.

### Aim

This systematic review aimed to investigate the effectiveness of in-hospital simulation-based training as a learning and teaching method to develop qualified healthcare teams’ human factor skills.

## Methods

The AMSTAR 2-criteria (A MeaSurement Tool to Assess systematic Reviews) were used to prepare the review [35]. The review report follows the Preferred Reporting Items for Systematic Reviews and Meta-Analysis (PRISMA) statement [36]. Details of the protocol were registered in the International Prospective Register of Systematic Reviews (PROSPERO) [37] (record ID: CRD42021118670).

### Search strategy

SPICE (Setting, Perspective/population, Intervention, Comparison and Evaluation) [38], an alternative to the qualitative conceptualising model PICO [39], provided a framework for the formulation of questions, keywords and the search process. The SPICE elements were outlined: Setting = in-hospital healthcare specialisms and units; Population = all authorised qualified clinical healthcare personnel, apart from dentists and pharmacologists; Intervention = using SBT to teach HFS; Comparison = SBT compared to classroom teaching or no training; and Evaluation = improvements in the personnel’s HFS.

Boolean operators were used, combining keywords and blocks. Furthermore, the databases’ unique thesauri, truncation, phrase searches and proximity searches were included. An experienced information specialist (author TFF) optimised the search. Publications in English, Danish, Norwegian and Swedish were deemed eligible.

The following databases were searched: CINAHL (EBSCO), Cochrane Library, EMBASE™ (OVID), ERIC (EBSCO), MEDLINE® (OVID), PsycINFO (OVID), SCOPUS and Teacher Reference Centre (EBSCO), September 28^th^, 2021. Search histories are available in Supplement A.

### Study selection and critical appraisal

Covidence [40], a screening and data extraction tool, was used in the study selection process. Except for reviews, research protocols and conference abstracts, all study design and publication types were included. Authors LA, MLH and ABN individually performed the title and abstract screening using a standardised pre-piloted guide of inclusion and exclusion criteria (Table 1). Communication with patients or relatives and virtual reality were excluded as the focus was on the performance of qualified healthcare teams. Studies using role-play were excluded because some team members role-play it does not resemble the everyday practice where every team member interacts due to the situation and competencies. The role-playing personnel has a role and a script and therefore only acts if given a significant task.

**Table 1:**
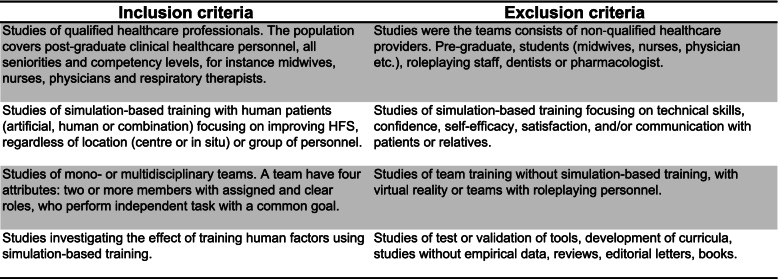
Inclusion and exclusion criteria

Conflicts were resolved through dialogue. LA subsequently selected eligible studies for inclusion by full-text reading, and, in cases of doubt, the consensus was achieved by consulting the authors MLH, ABN, LH and SVS. Each study was scrutinised for validity, reliability, generalisability and replicability of the results using the Critical Appraisal Skills Programme checklists (CASP) [41], Mixed Methods Appraisal Tool (MMAT) [42] or Critical Appraisal of a Survey [43]. The studies were labelled with either a high, medium or low-reliability rating for use in the analysis of effectiveness.

### The analysis process

Content analysis [44, 45] was used to assess the effectiveness. Content analysis is a systematic and objective research method that enables qualitative and quantitative content analysis. Stemler’s inductive technique was used to analyse the content. From open coding to creating themes and abstraction [44]. The following topics framed the content analysis: *characteristics*, *target population*, *HFS focus*, *intervention type and content*, *type of assessment*, *outcome*, *results and limitations*, *summaries of intervention effects* for each study. Due to the variation of the included study types, all assessments and methods were analysed and categorised. Every theme was verified and, where necessary, revised or split into two.

### Ethical consideration

Ethical approval was not deemed necessary because data was from previously published studies, but the study meet(s) the claims of the Helsinki Declaration [46].

## Results

**Fig. 1:**
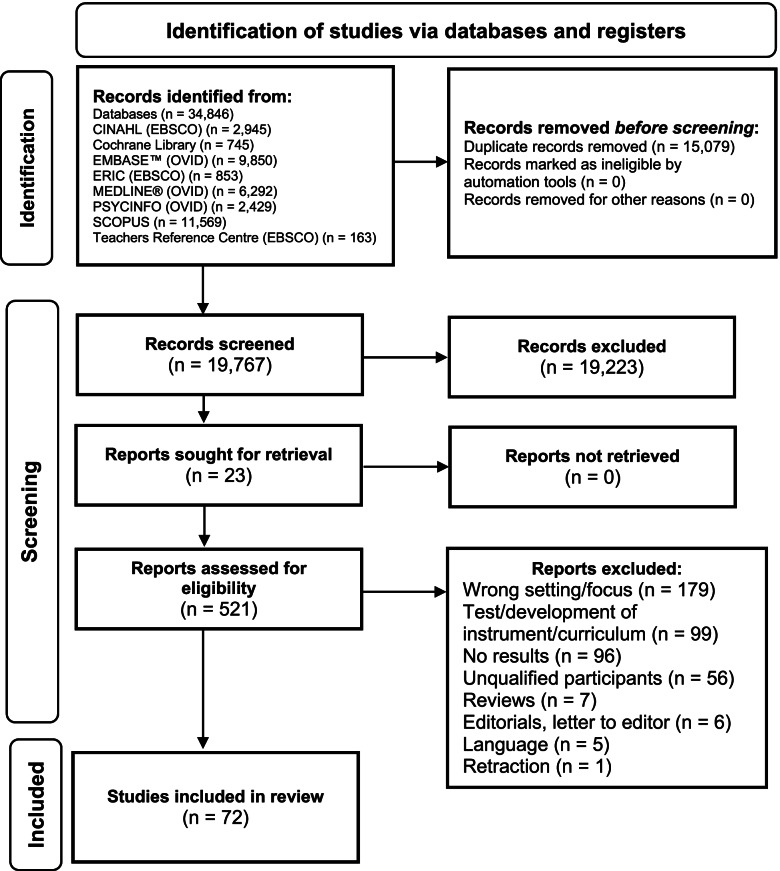
PRISMA flow diagram of the screening and selection process

The initial search identified 34,846 publications, representing 19,767 unique studies, after removing duplicates. After title and abstract screening, 521 studies were identified for full-text screening, of which 72 were included for data extraction and synthesis. This process is shown in the PRISMA flow diagram (Fig. 1).

### Result of quality assessment of included studies

The included studies were of varying quality, as shown in Table 2. The assessment included factors, such as unsuitable assessments methods, unclear selection methods, and uneven weighting of HFS and TS, favouring TS in assessing effectiveness. No studies were excluded following the quality assessment; however, it was used as an indicator of validity and reliability of the effectiveness of HFS training.

**Table 2:**
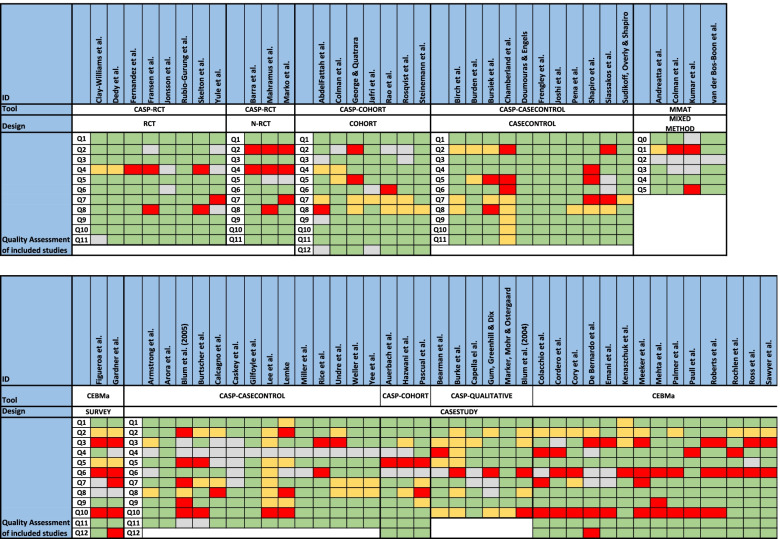
Quality assessment of 72 studies included in a systematic review of The effectiveness of improving healthcare teams’ human factor skills using simulation-based training. Green = Yes, Red = No, Grey = Can’t tell, Yellow = Not relevant, Q = Question

### Study characteristics

Included studies were published between 2004 and 2021 and were conducted mostly (*n* = 70) in Western countries. The 72 studies used 51 different assessment methods to measure the outcome of the team training interventions, including pre-tests, peri-tests and post-tests, (un)blinded ratings, self-assessments, surveys and interviews. The methods were validated (*n* = 30), non-validated or no information about validation (*n* = 14) and modified versions of validated (*n* = 9) instrument. The studies reported SBT settings such as simulation centres (*n* = 36), in-situ training (*n* = 24) and the use of both centre and in-situ training (*n* = 7). A broad variation was seen in the size and range of the studies (*n* = 7 to 675 participants) and represented SBT within seven different in-hospital medical specialisms: anaesthesiology (*n* = 7), emergency medicine (*n* = 20), intensive care (*n* = 9), internal medicine (*n* = 2), obstetrics (*n* = 12), paediatrics (*n* = 6) and surgery (*n* = 15). A range of teaching methods were used: SBT (*n* = 30); SBT and didactics (*n* = 34); SBT, didactics and workshops (*n* = 6); and SBT and workshops (*n* = 1).

The courses in the included studies were mostly stand-alone (*n* = 51), meaning not part of formal educational (*n* = 18) progress. The participants were either voluntary (*n* = 35), mandatory (*n* = 16), randomly selected participants (*n* = 9) or not stated (*n* = 12). Participants trained one or more HFS: communication, coordination, decision-making, followership, leadership, situational awareness, task management or teamwork.

Team size varied from two to twenty members, typically training in teams of two to five members. Two-thirds of the studies were of multidisciplinary teams (*n* = 47). Midwives, nurses and physicians were the most common participants, but 13 different disciplines participated. Mono-disciplinary SBT was seen in 20 studies; physicians (*n* = 18) were primarily trained separately from other qualified personnel. An extracted summary of included studies is shown in Table 3, and the whole summary is available in Supplement B.

**Table 3:**
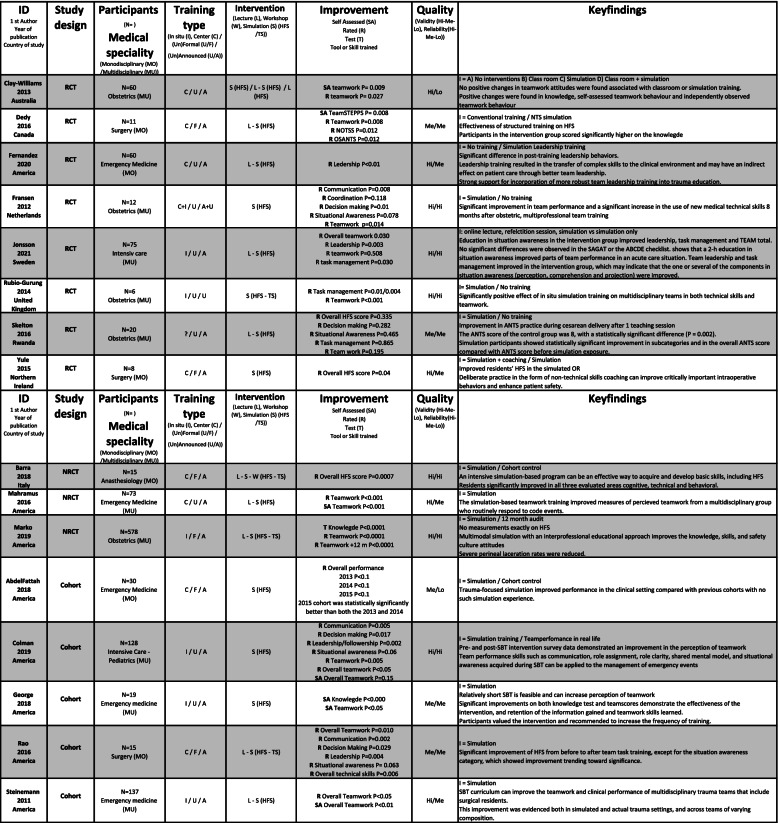

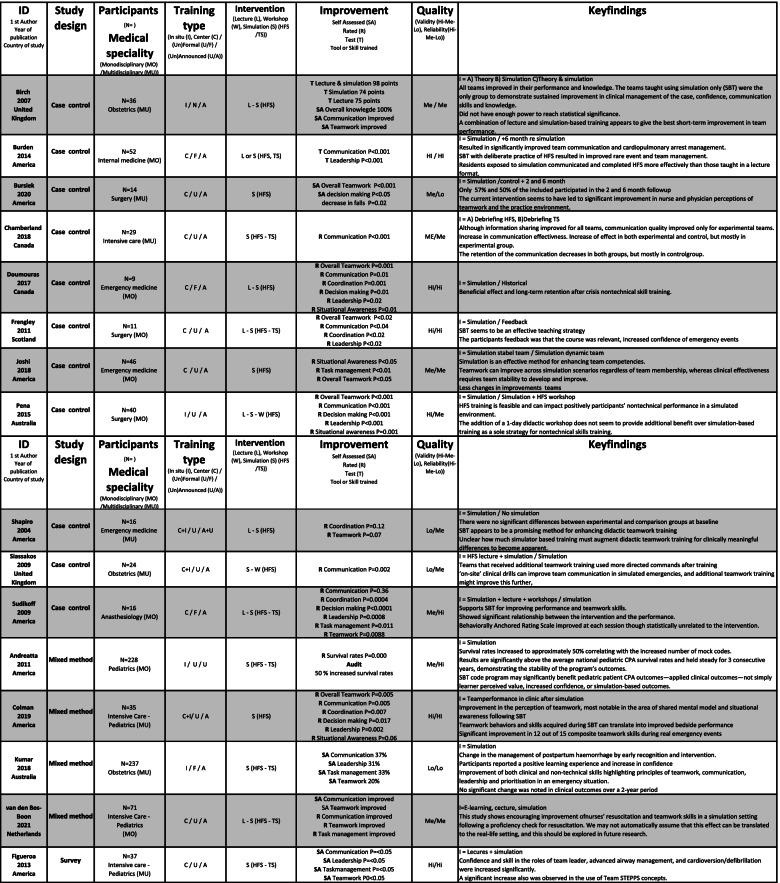

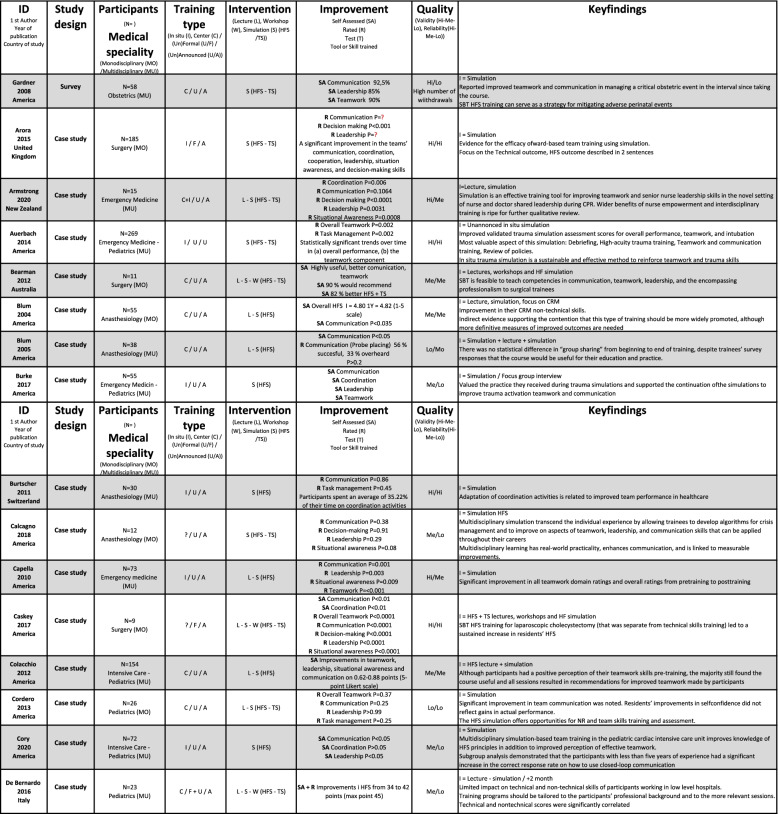

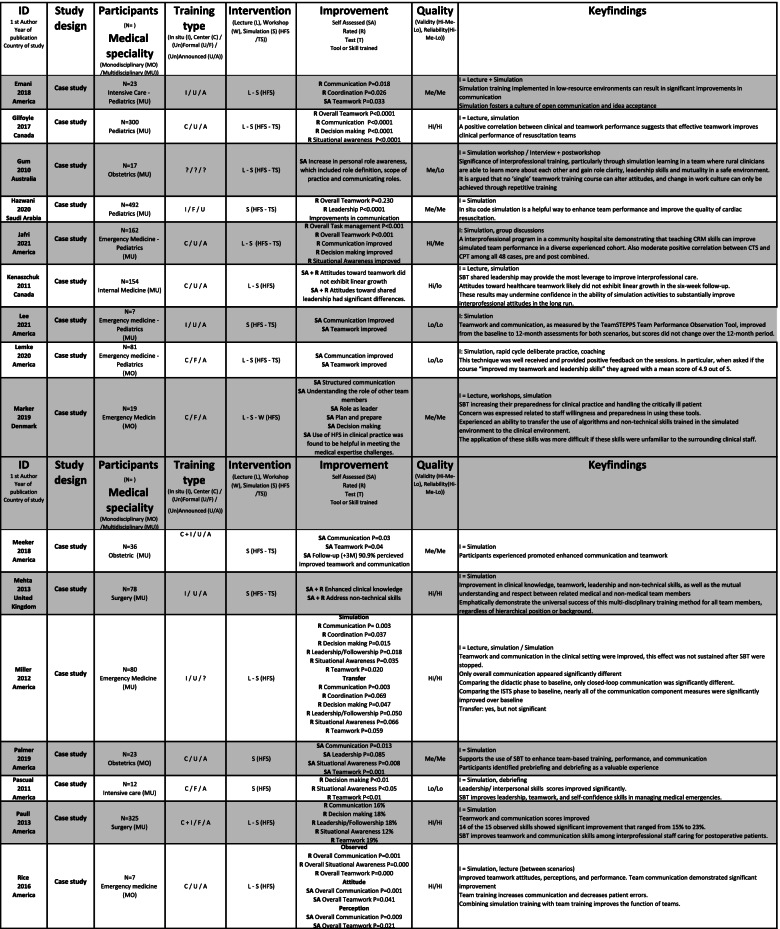

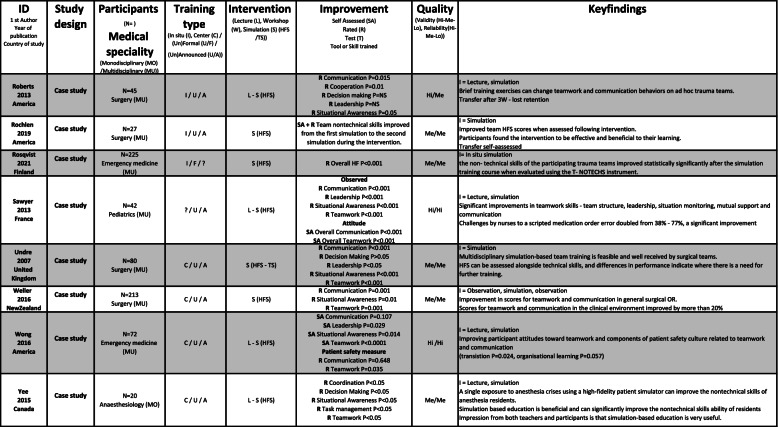
Extracted summary of studies included in a systematic review of The effectiveness of improving healthcare teams’ human factor skills using simulation-based training. The full summary of included studies is available in Supplement B

### Content analysis

The content analysis identified four recurring themes: (1) Training HFS in qualified teams, (2) assessment of HFS, (3) combined teaching methods and (4) retention and transfer of skills. These themes will be elaborated on below.

#### Training HFS in qualified healthcare teams

The vast majority (*n* = 65) of the studies concluded that SBT could develop qualified teams using HFS. In two-thirds of the studies, HFS as the sole focus of the training were seen and associated with enhanced effectiveness [13, 47–73]. These studies were mainly conducted in simulation centres, with smaller teams (*n* = 2–8 members), and the SBT-courses were announced. It is a significant result that HFS usually are trained together with TS, and when trained on its own, it is taught in centres rather than in situ and minor teams. Most of the 27 studies (*n* = 22) used validated assessment methods and performed debriefing (*n* = 24) immediately after every SBT scenario. Nevertheless, Emani et al. [60] and Jafri et al. [74] show a correlation between TS scores and HFS scores, which emphasises that the effect of SBT is evident when HFS is trained solely in combination with other competencies. Studies of multi-disciplinary training (*n* = 47) [13, 48, 53, 56, 58–64, 66–69, 71–102] were generally associated with greater effectiveness than mono-disciplinary training, perhaps because multi-disciplinary training better reflects everyday clinical practice.

Three studies showed potential effect [71, 93, 99], concluding that SBT is a promising tool to train HFS but that more applicable assessment methods are needed. Only two studies did not show effect [85, 98]; they mention positive selection bias because high numbers of participants withdrew, along with methodological problems and lack of assessment methods as possible causes of the non-effect result.

The trainees were mainly personnel from acute or high-intensity medical departments, and nearly all the trained situations involved acute life and death situations. Only four studies [68, 74, 93, 100] trained HFS in day-to-day work, such as reducing falls, ethical issues, delirium, the busy ward and caring for older patients and relatives. A paediatric focus was found in 25 SBT studies, in anaesthesiology, intensive care and obstetrics [13, 56, 60, 61, 72, 74–77, 80, 81, 83, 86, 88, 90, 91, 98, 102–109]. In total, 3251 of the participants were trained in acute paediatric scenarios. HFS during resuscitation (*n* = 20) was the second most trained situation [10, 13, 49, 52, 53, 59, 61, 62, 65, 72, 76, 78, 87, 89–91, 101, 104–108, 110], involving 1887 personnel. This illustrates that acute and high-intensity situations are the main focus of SBT concerning teams' HFS. Common to these training situations are available algorithms and checklists of the TS or HFS (e.g. acute caesarean, cardiopulmonary resuscitation, Crisis Resource Management), which facilitate a form of corrective actions. However, compliance with checklists and training algorithms does not cover the dynamics of HFS. Checklists and algorithms are task-oriented (check of rhythm, request read-back) that differ from the nature of HFS, which are social and cognitive processes within environmental and organisational frames. These task-oriented approaches increase the risk of changing the focus from the all-around focus to the tasks themselves. This could be why the focus on TS overtakes the focus on HFS in some of the studies, for instance, in Arora et al. and Siassakos et al. [99, 111].

This demonstrates that SBT increases the HFS among qualified teams, but due to the lack of high-quality studies using similar assessment tools, the level of effectiveness was not established.

#### Assessment of HFS

The studies lack an adequate description of how HFS refinements should be assessed. Existing HFS assessment tools are insufficient, which was emphasised in 28 studies [49, 55, 58, 61, 64, 65, 68, 71, 75, 78, 80, 81, 84, 85, 87, 89, 95, 96, 98, 99, 103, 107, 111–115]. Assessment methods (*n* = 51) spanned quantitative, qualitative and mixed methods, validated and non-validated methods, rating behavioural markers, rating via checklists, interviews, self-assessments, passing probes of information, measuring time and evaluation of reported experiences. Even though the studies used different assessment methods, they concluded that HFS enhanced among the participants. In 68 studies, HFS was considered to have improved and a significant development in HFS as a result of SBT was shown in 33 studies [10, 47–49, 51–56, 59, 60, 62, 64, 65, 72–77, 79, 80, 83, 87, 90, 100, 101, 104, 107, 108, 114, 116]. In conclusion, SBT can refine HFS.

The primary challenge in assessing HFS was a lack of definitions for HFS and insufficient coverage of many different HFS. HFS were, as mentioned, undefined or broadly described in several studies, or the assessment was unfit for HFS, such as measuring the time from the outset of a procedure to a specific action or treatment [13, 51, 61, 83, 89]. For instance, the increased time could also be due to improvements in the TS and not the HFS. HFS training associated with specific behaviour markers were the most successful assessment [10, 49, 54, 59, 65, 72, 73, 79, 101, 102, 114]. Five tools generally inspired the methods used: crisis resource management [117, 118]; Kirkpatrick Model: Four Levels of Learning Evaluation [119]; Mayo High-Performance Teamwork Scale [120]; Ottawa Global Rating Scale [121]; and TeamSTEPPS® [122].

The rating of markers was either blinded or unblinded by internal or external faculty or assessed by the participants themselves. Self-assessments were used in 31 studies. Self-assessment were used in combination with other methods in 18 studies [47, 53, 57, 60, 65, 67, 68, 72, 78, 81, 85, 88, 93, 95, 97, 98, 108, 116], whereas 13 studies used self-assessment as the only method [82–84, 87, 92, 94, 100, 102, 105, 107, 109, 110, 112]. There are inherent challenges in using rating and self-assessments because assessors must be congruent and unbiased, and participants tend to overrate their performance and therefore, the method has been proven unreliable [123, 124]. Some studies (*n* = 21) used video recording and blinded assessors [47, 48, 54, 58, 60, 61, 63, 66, 70, 71, 74, 76, 89, 91, 98, 99, 103, 106, 108, 111, 114], which increased the validity of the ratings; because the assessors’ could rewind the video and review the situation multiple times. Other studies rated participants in real-time, which challenged the assessors’ ability to simultaneously watch, listen and rate [10, 49–51, 53, 57, 59, 62–65, 67, 68, 72, 73, 75, 77–79, 81, 85, 93, 96, 101, 107, 115, 125].

The most frequently trained HFS were communication, leadership and teamwork. The specification of the trained HFS were described in various ways. Eleven studies [10, 13, 54, 69, 71, 98, 100, 101, 103, 114, 115] described HFS with behaviour markers, attitudes or as a definition of the chosen HFS, while others (*n* = 15) only mentioned the HFS in broad indefinite terms such as communication or teamwork [49, 57, 58, 63, 73, 76, 79, 85, 88, 89, 102, 106, 108, 109, 112]. Communication and teamwork were the two most trained HFS.

Communication and teamwork are both broad terms. Communication and teamwork are not isolated and unequivocal tasks; they depend on and influence each other, like most HFS. The purpose of outlining and dividing the tasks into behaviour markers is to simplify a complex clinical situation, i.e. highlight easily recognisable behaviour for the participants, making it easier to acquire and develop skills [118, 126]. The studies that described HFS using either behaviour markers or attitudes succeeded to a greater extent in assessing HFS and developments than those that described HFS in broad, indefinite terms. It is difficult to determine and report the effect of training when the focus is on general terms such as communication and teamwork without a definition or level of detail. It is not possible to distinguish between teamwork/communication and cognition. While communication and teamwork are often immediately recognisable and valid interpretations for training personnel, they are high-level concepts difficult to rate to assessors. Maybe because you know it when you experience it but not always when you see it. However, the studies that reflected on the use of high-level concepts and worked to specify these in behaviour markers achieved greater internal validity along with assessed facts, due to the increased transparency [10, 13, 47, 48, 50, 52–55, 65–67, 69–72, 74, 75, 77, 78, 96–98, 100, 101, 103, 107, 114, 116].

#### They combined teaching methods

Significant effects on HFS were observed in 32 studies that combined SBT with didactics and workshops, compared to 12 that just trained SBT. The impact on qualified teams’ use of HFS was evident, regardless of whether SBT was combined with didactics and workshops or training HFS on their own or in combination with TS. HFS training was combined with TS training in 30 of the studies, of which 19 showed a significant effect on one or more HFS, equalling 48 of all the included studies. Thus, it appears that the studies in which HFS training was separate from TS training resulted in the most significant improvements in the teams’ use of HFS.

The studies that combined HFS and TS training tended to focus more on TS. For instance, Burden et al. and Siassakos et al. covered the results of HFS training with only a few sentences [99, 125], and Hazwani et al. asserted that a refined time to first medicine infusion in cardiopulmonary resuscitation training was because of an enhancement in teamwork [13].

### Retention and transfer of skills

Retention or transfer of HFS was explored in 21 of the studies. The retention of HFS were measured from participants’ knowledge, self-assessment, audits and patient outcome. Transfer of enhanced HFS are identified in 20 studies, but in two of these [79, 104], the authors identify transfer due to developed TS. The researchers argue that improved TS and time decrease in accomplishing the procedure are due to an increase in HFS skills. Roberts et al. find a transfer of HFS, but with low retention over time [66]. The transfer of HFS was measured as a decrease in adverse events and improved patient outcomes in six studies [49, 59, 79, 95, 97, 104].

## Discussion

This systematic review demonstrates that SBT is a successful learning tool to improve HFS in-hospital healthcare settings. Unfortunately, we were unable to show the effect level due to the use of all the different assessment tools. More research is required to increase knowledge about the transfer of competencies to daily clinical practice, examining why many studies use non-validated assessment strategies and the barriers to training HFS. While HFS are widely taught, there are gaps in the literature regarding efficacy assessment. There is a need for more long-term studies and studies about how we translate assessment of skills to clinical work. However, there is a lack of knowledge about the transfer and retention of the HFS developed, from SBT to actual competencies in clinical practice. The culture of viewing HFS as innate and complicated to train could be one of the obstacles.

Although this review revealed support for training HFS in the clinical setting using SBT, there is a lack of agreement on which tools are best to assess HFS. There are gaps in the literature regarding the assessment of the HFS. More research and consensus on how we assess HFS is needed before the level of effectiveness can be estimated. All assessment methods in SBT should be supported by valid evidence. Several instruments are designed to evaluate the effect of HFS skills through SBT. Still, this review shows that the existing assessment methods are not solid enough to establish consensus on the way HFS are assessed. Although tools exist to assess HFS, methods to study communication and other team-related processes are far from being standardised, making comparison challenging. This raises new questions about training HFS and future directions for research.

Cognition is an emergent property of the situation and environment. Knowledge, perceived facts, understanding and predictions within each team member’s mind interact with displayed information, cues and devices in the environment to affect decision-making and situational awareness. Recurrent exposure to these factors can lead to personal, team and institutional learning. Furthermore, the environment can be modified and redesigned to support the team’s improved performance and safety. Cognition is thus an individual and shared mental process within the team in all situations [127–129]. Therefore, it is essential to add social, cognitive, environmental and technology markers to the teaching/learning situations if the goal is to enhance the teams’ HFS or redesign the environment to increase patient safety. Nevertheless, 43% of the studies show significant effectiveness in refining HFS using SBT, and 92% show some effectiveness. This means that, regardless of multiple assessment methods, this review offers a significant or improved effect of HFS using SBT, and the outcome was relatively homogeneous—HFS improves using SBT. A meta-analysis by Salas et al. concludes that team training is a useful intervention with a moderate, positive effect on team processes [130]. This adds to the reliability of the present review. Therefore, the differences among the methods in the included studies are not a weakness of the research but rather a strength for the results. On the other hand, it makes the results inconsistent because of the lack of comparability. More research and effort towards a consensus on assessing human factor skills in the medical simulation society are requested.

The review also demonstrates that studies in which HFS was trained alone had a more significant effect than those focused on both HFS and TS. However, although the increase of HFS was lower in combined TS and HFS training, HFS was still enhanced in most studies. In SBT research, HFS are often relegated to an add-on to develop procedures, algorithms and associated TS in specific settings. This may be for several reasons: everyday clinical situations involve both HFS and TS, trained together, or it is easier to measure technical outcomes. HFS often play a minor role in the conclusions drawn. In this way, TS “steal” the focus, and the focus is on solving the medical problem at hand (e.g. bleeding or anaphylaxis) rather than improving HFS, which generally are the cause of most adverse events [34]. HFS are unfortunately often understood as innate skills and not skills that can be trained and refined. HFS are not innate; they are generic and essential in reducing adverse events within healthcare and need to be qualified and trained just as seriously as technical skills and clinical procedures.

The high amount of studies from acute and high-intensity situations and the paediatric speciality shows that there is awareness of the need for training qualified personnel, that SBT is not only for the students and novices. The training mostly around algorithms is unclear and could be an exciting focus in future research. Nevertheless, the results also show that qualified teams mostly train situations where life is at stake. However, adverse events not only happens in highly acute situations but also in slow situations such as medication administration [131], receiving and transferring patients [132, 133] and development of sepsis [134]—all situations where teams interact. If healthcare teams are trained in everyday care, it might reflect everyday clinical practice and prevent or reduce future adverse events.

An interesting result is that the training teams mostly were 2–5 members, although critical care teams are more prominent in numerous places in the world. The reasons for this are unclear, but possible explanations include the expense of SBT and a high turnover of qualified healthcare personnel [135]. Moreover, the participants are often volunteers, and the likely absence of volunteers can explain.

It is important to understand learning holistically, integrating the individual, brain, body and surroundings [136]. All levels of education involve both physical and cognitive stimulations, and if the content is too vast, the learning decreases. The results suggest that focusing exclusively on HFS in SBT can lead to a deeper awareness of HFS's effect on patient safety among teams and, possibly consequently larger learning potential. However, further research will have to study to what degree HFS transfers to competence in clinical practice. The results show that SBT for HFS alone, combined with didactics and workshops may lead to the most significant improvement in teams’ HFS. This is substantiated by Maturana’s theory of suitable disturbances [137, 138], which deals with how disturbances should be moderated. If a disturbance is too big, the learners might lose attention, and if the disturbances are too small, the learners might not even notice. Accordingly, if TS and HFS are trained together, the educational disturbance to participants’ behaviour might be too massive for participants to engage with. However, the link to clinical practice is still underdeveloped.

## Conclusion

This systematic review demonstrates a strong indication that SBT is an effective learning tool to improve HFS in-hospital healthcare settings. However, HFS are inconsistently described, interpreted, taught and assessed and the lack of real-world assessment or follow-up makes the transfer to everyday practice challenging. This systematic review does not entirely answer if SBT improves HFS in qualified healthcare teams. Still, it highlights the gaps in the literature and underpins the necessity of increasing the focus on HFS or routine care in SBT to improve outcomes. There is a need for more long-term studies and studies about how we translate assessment of skills to clinical work. However, there is a lack of knowledge about the transfer and retention of the HFS developed, from SBT to actual competencies in clinical practice. The culture of viewing HFS as innate and complicated to train could be one of the obstacles. Healthcare, in general, must support the necessity and significance for HFS. Otherwise, the HFS will not be effectively transferred to everyday practice. Also, design issues such as positioning of the equipment, cognitive aids and process changes are needed to support ideal human performance such as not relying on memory or complex decision-making in complex time-pressed situations. More research is required to increase knowledge about the transfer of competencies to daily clinical practice, examining why many studies use non-validated assessment strategies and the barriers to training HFS.

### Limitations

A few limitations of this review need to be highlighted. Firstly, three authors screened a vast number of studies, but only the first author did a full-text reading and assessment of the included studies. This increases the possibility of selection bias and influences the internal validity and reliability. The bias was sought to be minimised by bringing any doubts about selected studies to the broader author group. Nevertheless, the intercoder reliability is inevitably affected when human coders are used in content analysis [139]. Secondly, the Hawthorne effect (behaviour alteration simply because HFS were studied) represents a possible bias [140]. Thirdly, 48% of the participants in the included studies courses were volunteers, but the results from volunteer studies do not deviate from the enhancement among mandatory participants. Nevertheless, the number of volunteers could lead to a positively biased result because they agreed to SBT as a learning method. Moreover, it is essential to point out that 20 of the included studies were from an emergency medicine setting, which can have influenced the results. A review focusing on HFS, in general, could have elucidated studies from other settings. Finally, the results may be affected by publication bias because studies with unfavourable results of SBT might not have been published, which could mean an endorsement of the results in the direction of a favourable analysis.

### Implications for practice

It is evident that SBT can improve qualified teams’ HFS. SBT is an effective learning tool for use with novices and experts, and with unqualified or qualified personnel. A change of focus is recommended for healthcare providers to train emergencies or rare situations and everyday non-emergency situations, such as admission to hospital, rounds, or the unprepared talk with next-in-kind in the hallway. This review shows that even qualified teams’ can develop their HFS significantly through SBT. Using SBT to train the healthcare personnel for everyday clinical practice are essential. Firstly, because the everyday routine takes up most of the performance tasks in the hospitals, the personnel are constantly in different forms of teamwork. Secondly, as learned from Safety II, it is necessary to enhance the ability to succeed (reduce adverse events) under varying conditions [141]. Thirdly, healthcare personnel are constantly interchangeably with new demands (e.g. professional, environmental and technical) to the personnel. Finally, yet significantly, the high degree of personnel turnover in healthcare affects the quality of care, a quality that the use of continual SBT can increase. If the personnel’s HFS are capable in everyday practice, they will in all probability be in acute and high-intensity situations.

All human interactions in hospitals need to be efficient and trained just as seriously as TS and clinical procedures because interactions are just as prone, if not more, to errors. Cultural, social and people skills, together termed HFS, are not innate and untrainable. Instead, they are generic and essential in reducing adverse events within healthcare and demands an increased focus on systematic multidisciplinary training of HFS among healthcare teams.

## Supplementary Information

**Additional file 1.** Supplement A–Searches.

**Additional file 2.** Supplement B–Results summary.

## Data Availability

The datasets used and analysed during the current study are available from the corresponding author on reasonable request.

## Abbreviations

AMSTAR-2: A measurement tool to assess systematic reviews–2^nd^ edition
ANTS: Anaesthetists' non-technical skills
CASP: Critical appraisal skills programme
CINAHL: Cumulative index of nursing and allied health literature (database)
COVID-19: Coronavirus disease 2019
COVIDENCE: An online tool that streamlines parts of the systematic review process
CRM: Crisis resource management
EBSCO: Elton B. Stephens company (online access)
EMBASE^TM^: Excerpta Medica database (database)
ERIC: Educational resources information center (database)
HFS: Human factor skills
MMAT: Mixed methods appraisal tool
MEDLINE: Medical literature analysis and retrieval system online (database)
NTS: Non-technical skills
Non-RCT: Non-randomized controlled trial
OVID: Part of the Wolters Kluwer group of companies (online access)
PICO: Problem/population, intervention, comparison, and outcome
PROSPERO: Prospective register of systematic reviews
PRISMA: Preferred reporting items for systematic reviews and meta-analyses
PsycINFO: Psychological information (database)
RCT: Randomized controlled trial
SCOPUS: Elsevier’s abstract and citation database
SBT: Simulation-based training
SPICE: Setting, perspective, intervention, comparison, and evaluation
TS: Technical skills

## Footnotes

1 Adverse events: an event that results in injury or risk of injury during health professional activity. The incident is unintentional and includes known and unknown events and errors that are not due to the patient's illness and that are either harmful or could have been harmful (near-accident).
